# Supplementary material for: Reliability of dynamic and isometric upper muscle strength testing in breast cancer survivors
Source: PeerJ. 2024 Jul 23;12:e17576. doi: 10.7717/peerj.17576 (PMC11276762; doi:10.7717/peerj.17576)
Supplement: Supplemental Information 1 [file peerj-12-17576-s001.docx]

Supplementary Material

**Reliability of Dynamic and Isometric upper muscle strength testing in Breast Cancer Survivors.**

**1 Supplementary Figures and Tables**

**1.1 Supplementary Figures and Tables – With or without lymphedema**

Supplementary material 1. Analysis of intra-day reliability and error of measurement on the bilateral isometric bench press in breast cancer survivors with lymphedema (N = 12) and without lymphedema (N = 20).

|  | Attempt 1  Mean (SD) | Attempt 2  Mean (SD) | Attempt 3  Mean (SD) | ICC  95%IC | CV %  95%IC | SEM  (SEM%) | MDC  (MDC%) |
| --- | --- | --- | --- | --- | --- | --- | --- |
| HGS lym - test |  |  |  |  |  |  |  |
| Treated side | 22.31 (6.48) | 22.02 (7.59) | 22.68 (6.85) | 0.986  (0.963 to 0.996) | 6.95  (4.05 to 9.85) | 0.81 (3.65) | 2.26 (10.11) |
| Non-treated side | 21.73 (5.72) | 22.40 (6.02) | 21.83 (5.27) | 0.983  (0.956 to 0.995) | 5.04  3.21 to 6.87 | 0.73 (3.31) | 2.02 (9.17) |
|  |  |  |  |  |  |  |  |
| HGS lym - retest |  |  |  |  |  |  |  |
| Treated side | 24.13 (6.04) | 24.06 (6.03) | 24.62 (5.98) | 0.994  (0.984 to 0.998) | 3.00  2.02 to 3.98 | 0.46 (1.91) | 1.28 (5.29) |
| Non-treated side | 23.49 (5.85) | 23.17 (5.53) | 23.34 (5.15) | 0.99  (0.975 to 0.997) | 3.69  2.42 to 4.96 | 0.55 (2.34) | 1.51 (6.49) |
|  |  |  |  |  |  |  |  |
| HGS nlym - test |  |  |  |  |  |  |  |
| Treated side | 26.15 (4.89) | 26.11 (4.95) | 26.03 (4.68) | 0.983  (0.964 to 0.993) | 3.78  2.86 to 4.70 | 0.62 (2.38) | 1.72 (6.59) |
| Non-treated side | 26.98 (3.51) | 27.38 (3.63) | 26.895 (3.43) | 0.955  (0.907 to 0.991) | 3.82  2.78 to 4.85 | 0.72 (2.65) | 1.99 (7.33) |
|  |  |  |  |  |  |  |  |
| HGS nlym -retest |  |  |  |  |  |  |  |
| Treated side | 26.32 (4.18) | 26.79 (4.02) | 26.85 (4.23) | 0.974  (0.945 to 0.989) | 3.56  2.10 to 5.02 | 0.65 (2.44) | 1.81 (6.77) |
| Non-treated side | 26.79 (3.81) | 27.08 (3.46) | 27.38 (3.88) | 0.973  (0.944 to 0.989) | 3.36  2.33 to 4.39 | 0.60 (2.20) | 1.65 (6.10) |
|  |  |  |  |  |  |  |  |
| BIBP lym - test |  |  |  |  |  |  |  |
| Treated side | 91.84 (20.46) | 93.14 (18.63) | 89.71 (19.28) | 0.978  (0.943 to 0.993) | 4.99  3.02 to 6.95 | 2.83 (3.09) | 7.84 (8.56) |
| Non-treated side | 97.63 (24.77) | 97.88 (25.15) | 93.25 (26.59) | 0.978  (0.943 to 0.993) | 5.79  3.32 to 8.26 | 3.71 (3.86) | 10.29 (10.69) |
|  |  |  |  |  |  |  |  |
| BIBP lym - retest |  |  |  |  |  |  |  |
| Treated side | 95.99 (21.39) | 97.06 (24.55) | 97.73 (24.82) | 0.986  (0.963 to 0.996) | 4.45  3.01 to 5.89 | 2.76 (2.84) | 7.64 (7.88) |
| Non-treated side | 95.84 (23.16) | 99.50 (22.51) | 98.92 (23.05) | 0.985  (0.96 to 0.99) | 4.35  2.52 to 6.18 | 2.77 (2.82) | 7.67 (7.82) |
|  |  |  |  |  |  |  |  |
| BIBP nlym - test |  |  |  |  |  |  |  |
| Treated side | 98.39 (22.53) | 97.34 (22.40) | 96.48 (22.28) | 0.984  (0.966 to 0.993) | 4.25  3.01 to 5.50 | 2.79 (2.86) | 7.73 (7.94) |
| Non-treated side | 108.35 (28.92) | 104.52 (26.43) | 104.30 (24.00) | 0.976  (0.95 to 0.99) | 5.04  3.56 to 6.52 | 4.02 (3.80) | 11.15 (10.54) |
|  |  |  |  |  |  |  |  |
| BIBP nlym -retest |  |  |  |  |  |  |  |
| Treated side | 103.10 (25.39) | 105.54 (27.31) | 104.75 (25.98) | 0.98  (0.958 to 0.991) | 4.90  3.58 to 6.22 | 3.64 (3.48) | 10.08 (9.65) |
| Non-treated side | 108.06 (23.16) | 107.10 (26.36) | 110.06 (25.94) | 0.968  (0.932 to 0.986) | 5.56  4.13 to 7.00 | 4.37 (4.03) | 12.10 (11.16) |
|  |  |  |  |  |  |  |  |
| BIBP lym |  |  |  |  |  |  |  |
| Summered - test | 189.47 (43.50) | 191.02 (41.93) | 182.97 (44.99) | 0.981  (0.95 to 0.994) | 4.40  1.96 to 6.83 | 5.89 (3.14) | 16.34 (8.70) |
| Summered - retest | 191.84 (42.66) | 196.56 (45.53) | 196.65 (46.27) | 0.99  (0.974 to 0.997) | 3.61  2.47 to 4.74 | 4.44 (2.28) | 12.31 (6.31) |
|  |  |  |  |  |  |  |  |
| BIBP nlym |  |  |  |  |  |  |  |
| Summered - test | 206.74 (49.83) | 201.87 (47.44) | 200.78 (44.44) | 0.984  (0.967 to 0.993) | 4.14  3.00 to 5.29 | 5.89 (2.90) | 16.33 (8.04) |
| Summered - retest | 211.16 (45.61) | 212.64 (51.26) | 214.81 (49.94) | 0.977  (0.953 to 0.99) | 4.59  3.30 to 5.89 | 7.26 (3.41) | 20.13 (9.46) |

HGS, handgrip strength; BIBP, bilateral isometric bench press; lym, lymphedema; nlym, non-lymphedema; *N*, Newton; SD, standard deviation; CV, coefficient of variation; ICC, intra-class coefficient correlation; CI, 95% confidence intervals; SEM, standard error of measurement; MDC, minimally detectable change.

Supplementary material 2. Analysis of test-retest reliability and error of measurement on upper body muscle strength testing in breast cancer survivors with lymphedema (N = 12) and without lymphedema (N = 20).

|  | Test  Mean (SD) | Retest  Mean (SD) | ICC  95%CI | CV (%)  95%CI | TEM (TEM%) | SEM (SEM%) | MDC (MDC%) |
| --- | --- | --- | --- | --- | --- | --- | --- |
| HGS – lym |  |  |  |  |  |  |  |
| Treated side | 22.34 (6.88) | 24.27 (5.98) | 0.934  (0.69 to 0.983) | 8.54  1.62 to 15.46 | 1.94 (8.33) | 1.64 (7.04) | 4.55 (19.51) |
| Non-treated side | 21.99 (5.58) | 23.34 (5.46) | 0.971  (0.722 to 0.994) | 5.75  3.16 to 8.35 | 0.95 (4.20) | 0.93 (4.09) | 2.57 (11.34) |
| HGS – nlym |  |  |  |  |  |  |  |
| Treated side | 26.10 (4.76) | 26.66 (4.04) | 0.95  (0.875 to 0.98) | 4.51  2.80 to 6.23 | 1.34 (5.09) | 0.98 (3.70) | 2.70 (10.26) |
| Non-treated side | 27.09 (3.38) | 27.08 (3.63) | 0.946  (0.863 to 0.979) | 3.39  2.18 to 4.60 | 1.15 (4.24) | 0.80 (2.97) | 2.23 (8.23) |
| BIBP – lym |  |  |  |  |  |  |  |
| Treated side | 91.56 (19.07) | 96.92 (23.29) | 0.892  (0.639 to 0.969) | 8.568  5.05 to 12.08 | 9.07 (9.62) | 6.90 (7.32) | 19.13 (20.30) |
| Non-treated side | 96.26 (25.03) | 98.09 (22.60) | 0.918  (0.718 to 0.976) | 6.99  2.12 to 11.86 | 9.53 (9.81) | 6.68 (6.88) | 18.52 (19.06) |
| BIBP – nlym |  |  |  |  |  |  |  |
| Treated side | 97.40 (22.05) | 104.46 (25.72) | 0.88  (0.673 to 0.954) | 8.73  5.70 to 11.76 | 10.35 (10.25) | 8.28 (8.21) | 22.96 (22.75) |
| Non-treated side | 105.72 (25.96) | 108.41 (24.41) | 0.86  (0.647 to 0.944) | 8.21  4.88 to 11.53 | 12.63 (11.80) | 9.32 (8.70) | 10.77 (24.13) |
| BIBP – lym |  |  |  |  |  |  |  |
| Summered | 187.82 (42.77) | 195.01 (44.43) | 0.92  (0.736 to 0.977) | 7.33  3.68 to 10.98 | 16.67 (8.71) | 12.11 (6.32) | 33.56 (17.53) |
| BIBP – nlym |  |  |  |  |  |  |  |
| Summered | 203.13 (46.59) | 212.87 (47.89) | 0.886  (0.716 to 0.955) | 7.50  4.44 to 10.56 | 20.85 (10.03) | 15.83 (7.61) | 43.89 (21.10) |
|  |  |  |  |  |  |  |  |
| BP 1-RM - lym | 23.58 (6.53) | 25.58 (5.47) | 0.94  (0.592 to 0.985) | 6.61  2.02 to 11.20 | 1.59 (6.49) | 1.46 (5.96) | 4.06 (16.51) |
| BP 1-RM - nlym | 22.25 (4.15) | 22.75 (3.71) | 0.977  (0.937 to 0.991) | 2.59  0.87 to 4.31 | 0.78 (3.46) | 0.59 (2.63) | 1.64 (7.28) |
|  |  |  |  |  |  |  |  |

HGS, handgrip strength; BIBP, bilateral isometric bench press; lym, lymphedema; nlym, non-lymphedema; *N*, Newton; SD, standard deviation; CV, coefficient of variation; ICC, intra-class coefficient correlation; CI, 95% confidence intervals; SEM, standard error of measurement; MDC, minimally detectable change.

Supplementary material Fig. 1. Bland-Altman plots of maximum muscular strength tests: HGS, handgrip strength; BIBP, bilateral isometric bench press; and, 1-RM, one repetition maximum in BCS with lymphedema.


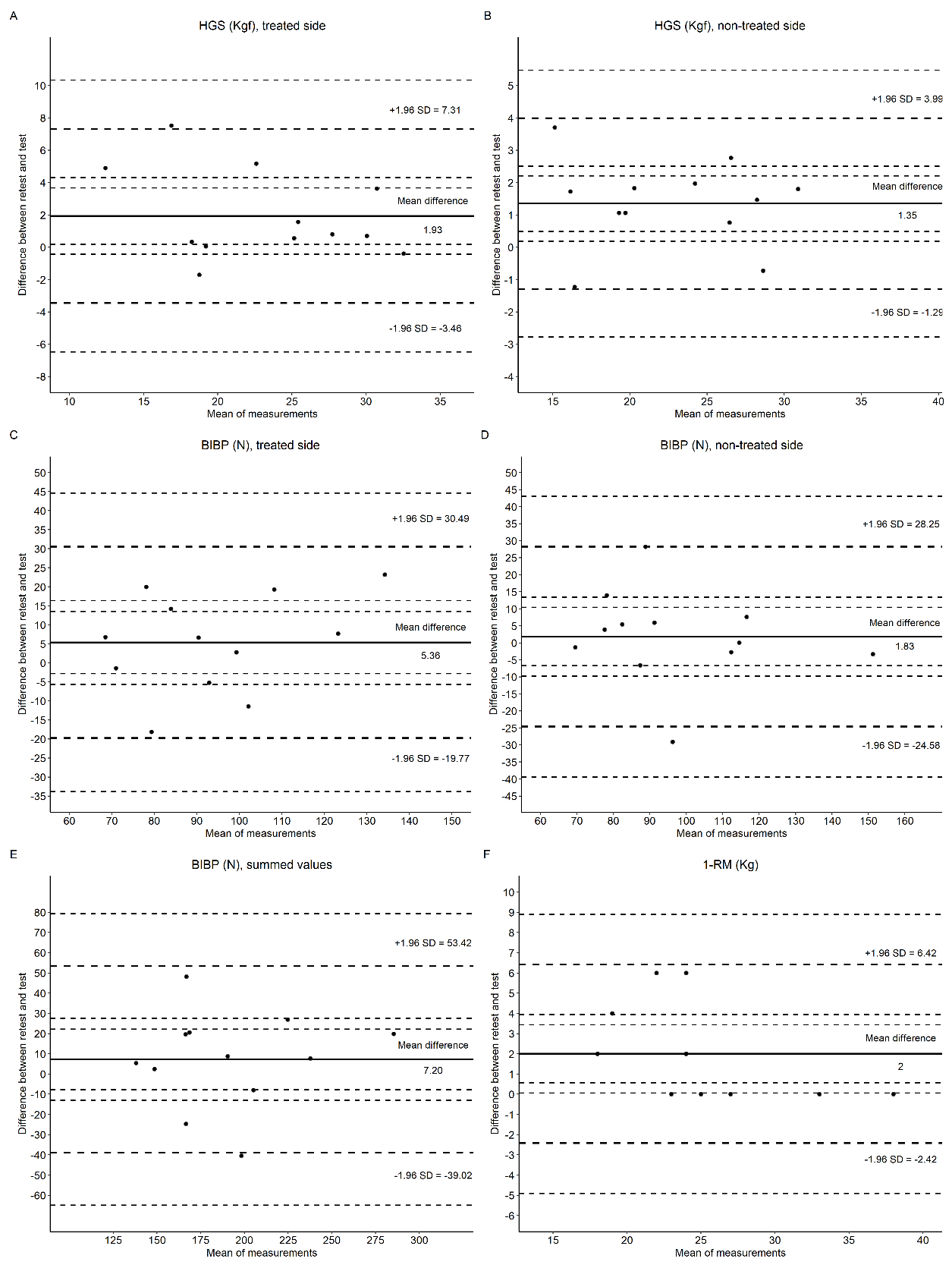


Leg. The dotted line (+1.96 SD and -1.96 SD) represents the limits of the agreement’s upper and lower boundaries. The continuous line (mean difference) on the center of the plot represents the systematic bias. The continuous line on the Y axis represents the mean difference between retest and test, and the X axis represents the mean of retest and test.

Supplementary material Fig. 2. Bland-Altman plots of maximum muscular strength tests: HGS, handgrip strength; BIBP, bilateral isometric bench press; and, 1-RM, one repetition maximum in BCS without lymphedema.


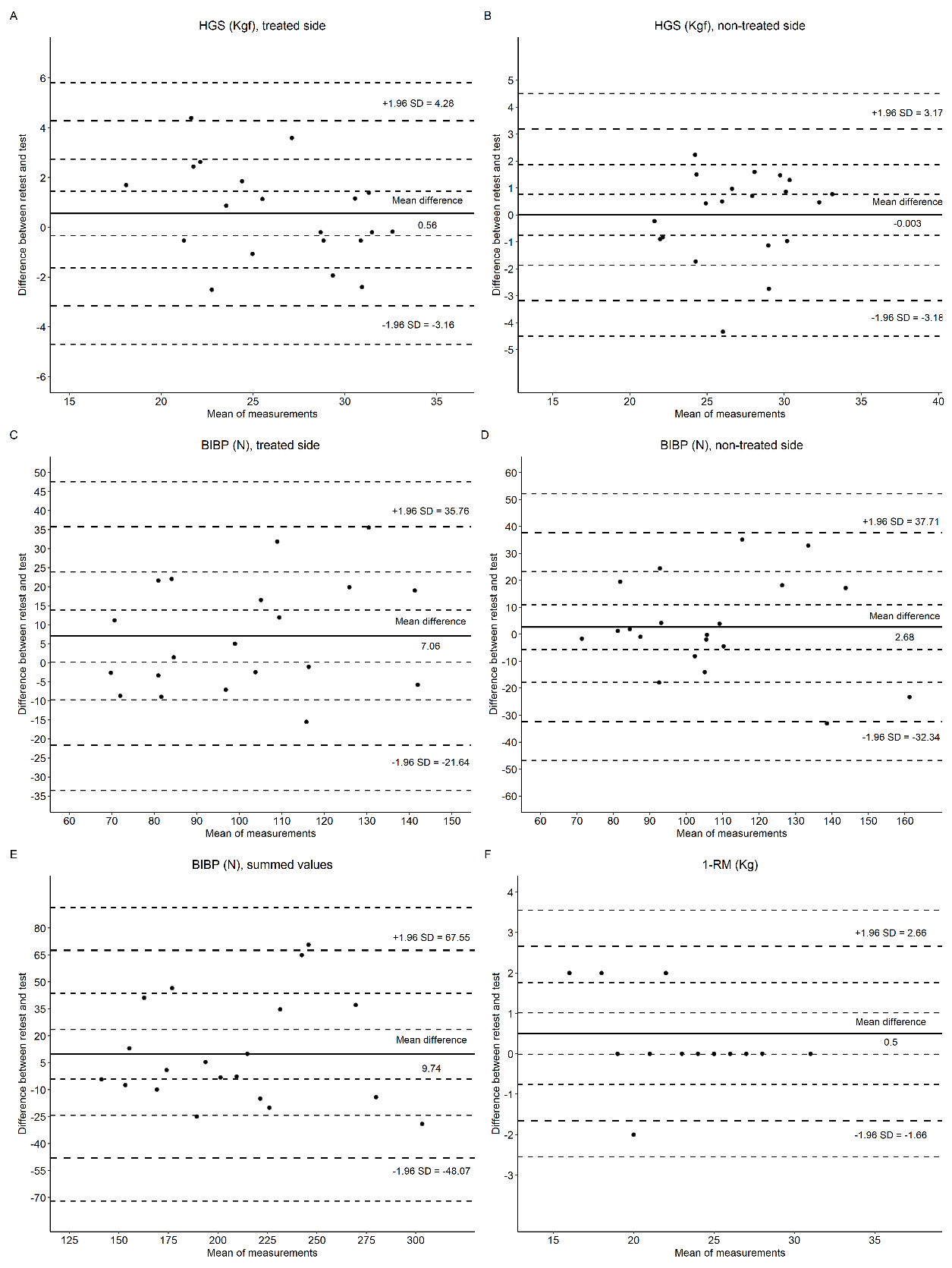


Leg. The dotted line (+1.96 SD and -1.96 SD) represents the limits of the agreement’s upper and lower boundaries. The continuous line (mean difference) on the center of the plot represents the systematic bias. The continuous line on the Y axis represents the mean difference between retest and test, and the X axis represents the mean of retest and test.

**1.2 Supplementary Figures and Tables – With or without mastectomy**

Supplementary material 3. Analysis of intra-day reliability and error of measurement on upper body muscle strength testing in breast cancer survivors with mastectomy (N = 15) and without mastectomy (N = 17).

|  | Attempt 1  Mean (SD) | Attempt 2  Mean (SD) | Attempt 3  Mean (SD) | ICC  95%IC | CV %  95%IC | SEM  (SEM%) | MDC  (MDC%) |
| --- | --- | --- | --- | --- | --- | --- | --- |
| HGS mastec - test |  |  |  |  |  |  |  |
| Treated side | 25.85 (6.043) | 25.75 (5.74) | 25.89 (5.33) | 0.99  0.976 to 0.996 | 3.65  2.13 to 5.15 | 0.56 (2.19) | 1.57 (6.07) |
| Non-treated side | 25.79 (4.09) | 26.17 (3.59) | 25.53 (4.04) | 0.981  0.956 to 0.993 | 3.39  2.63 to 4.15 | 0.53 (2.05) | 1.47 (5.70) |
|  |  |  |  |  |  |  |  |
| HGS mastec - retest |  |  |  |  |  |  |  |
| Treated side | 26.31 (4.52) | 26.90 (4.49) | 26.99 (4.51) | 0.987  0.968 to 0.995 | 3.11  2.01 to 4.20 | 0.51 (1.90) | 1.41 (5.26) |
| Non-treated side | 26.04 (4.06) | 25.99 (3.92) | 26.33 (4.41) | 0.983  0.956 to 0.994 | 3.24  2.18 to 4.230 | 0.53 (2.03) | 1.47 (5.63) |
|  |  |  |  |  |  |  |  |
| HGS nmastec - test |  |  |  |  |  |  |  |
| Treated side | 23.71 (5.47) | 23.55 (6.73) | 23.79 (6.03) | 0.982  0.959 to 0.993 | 6.13  4.21 to 8.06 | 0.80 (3.39) | 2.23 (9.40) |
| Non-treated side | 24.32 (5.86) | 24.94 (6.34) | 24.52 (5.50) | 0.978  0.954 to 0.991 | 5.05  3.549 to 6.61 | 0.86 (3.49) | 2.38 (9.66) |
|  |  |  |  |  |  |  |  |
| HGS nmastec -retest |  |  |  |  |  |  |  |
| Treated side | 24.79 (5.39) | 24.77 (5.27) | 25.15 (5.35) | 0.985  0.966 to 0.994 | 3.56  1.95 to 5.18 | 0.64 (2.58) | 1.78 (7.16) |
| Non-treated side | 25.12 (5.57) | 25.36 (5.36) | 25.145 (5.14) | 0.987  0.97 to 0.995 | 3.69  2.52 to 4.87 | 0.60 (2.438) | 1.67 (6.60) |
|  |  |  |  |  |  |  |  |
| BIBP mastec - test |  |  |  |  |  |  |  |
| Treated side | 99.26 (18.43) | 97.17 (18.24) | 96.03 (17.60) | 0.975  0.942 to 0.991 | 4.14  2.71 to 5.58 | 2.80 (2.87) | 7.75 (7.95) |
| Non-treated side | 107.29 (28.48) | 105.17 (25.19) | 101.66 (22.91) | 0.975  0.94 to 0.991 | 4.81  3.17 to 6.46 | 3.97 (3.79) | 10.99 (10.50) |
|  |  |  |  |  |  |  |  |
| BIBP mastec - retest |  |  |  |  |  |  |  |
| Treated side | 98.36 (20.63) | 101.31 (24.41) | 99.96 (21.62) | 0.967  0.931 to 0.989 | 4.87  2.99 to 6.74 | 3.75 (3.75) | 10.39 (10.40) |
| Non-treated side | 104.47 (25.08) | 104.79 (25.48) | 104.67 (24.71) | 0.967  0.92 to 0.988 | 5.79  4.00 to 7.58 | 4.40 (4.21) | 12.21 (11.67) |
|  |  |  |  |  |  |  |  |
| BIBP nmastec - test |  |  |  |  |  |  |  |
| Treated side | 93.00 (24.38) | 94.53 (23.41) | 92.09 (24.25) | 0.986  0.968 to 0.994 | 4.87  3.31 to 6.41 | 2.80 (3.00) | 7.76 (8.33) |
| Non-treated side | 101.73 (27.28) | 99.26 (26.68) | 98.83 (27.64) | 0.979  0.954 to 0.992 | 5.76  3.81 to 7.72 | 3.86 (3.86) | 10.71 (10.71) |
|  |  |  |  |  |  |  |  |
| BIBP nmastec -retest |  |  |  |  |  |  |  |
| Treated side | 102.27 (26.90) | 103.28 (27.56) | 104.02 (28.83) | 0.989  (0.976 to 0.995) | 4.60  3.70 to 5.51 | 2.91 (2.82) | 8.06 (7.81) |
| Non-treated side | 102.60 (22.88) | 103.77 (25.15) | 106.95 (26.18) | 0.982  (0.958 to 0.993) | 4.50  3.08 to 5.92 | 3.327 (3.13) | 9.06 (8.68) |
|  |  |  |  |  |  |  |  |
| BIBP mastec |  |  |  |  |  |  |  |
| Summered - test | 206.55 (45.91) | 202.734 (42.67) | 197.69 (39.81) | 0.98  0.951 to 0.993 | 3.97  2.46. to 5.47 | 5.96 (2.95) | 16.52 (8.17) |
| Summered - retest | 202.83 (43.93) | 206.10 (48.27) | 204.63 (44.66) | 0.974  0.938 to 0.99 | 4.75  3.07 to 6.42 | 7.16 (3.50) | 19.86 (9.71) |
|  |  |  |  |  |  |  |  |
| BIBP nmastec |  |  |  |  |  |  |  |
| Summered - test | 194.72 (49.76) | 193.79 (48.02) | 190.93 (49.76) | 0.986  0.968 to 0.994 | 4.47  2.79 to 6.15 | 5.74 (2.97) | 15.90 (8.23) |
| Summered - retest | 204.87 (46.96) | 207.06 (51.26) | 210.97 (53.13) | 0.989  0.975 to 0.996 | 3.76  2.82 to 4.69 | 5.47 (2.63) | 15.17 (7.31) |

HGS, handgrip strength; BIBP, bilateral isometric bench press; *N*, Newton; SD, standard deviation; CV, coefficient of variation; ICC, intra-class coefficient correlation; CI, 95% confidence intervals; SEM, standard error of measurement; MDC, minimally detectable change.

Supplementary material 4. Analysis of test-retest reliability and error of measurement on upper body muscle strength testing in breast cancer survivors with mastectomy (N = 15) and without mastectomy (N = 17).

|  | Test  Mean (SD) | Retest  Mean (SD) | ICC  95%CI | CV (%)  95%CI | TEM (TEM%) | SEM (SEM%) | MDC (MDC%) |
| --- | --- | --- | --- | --- | --- | --- | --- |
| HGS – mastec |  |  |  |  |  |  |  |
| Treated side | 25.83 (5.65) | 26.73 (4.45) | 0.944  0.832 to 0.981 | 5.28  1.01 to 9.55 | 1.60 (6.07) | 1.19 (4.52) | 3.29 (12.53) |
| Non-treated side | 25.82 (3.85) | 26.09 (4.07) | 0.954  0.866 to 0.985 | 3.90  2.49 to 5.431 | 1.19 (4.59) | 0.83 (3.22) | 2.31 (8.92) |
| HGS – nmastec |  |  |  |  |  |  |  |
| Treated side | 23.68 (5.98) | 24.90 (5.25) | 0.943  0.825 to 0.98 | 6.40  2.86 to 9.93 | 1.71 (7.03) | 1.33 (5.48) | 3.69 (15.20) |
| Non-treated side | 24.59 (5.78) | 25.31 (5.28) | 0.975  0.928 to 0.991 | 4.57  2.63 to 6.50 | 1.15 (4.60) | 0.86 (3.46) | 2.40 (9.60) |
| BIBP – mastec |  |  |  |  |  |  |  |
| Treated side | 97.48 (17.68) | 99.88 (21.64) | 0.882  0.653 to 0.96 | 8.19  5.43 to 10.795 | 9.20 (9.32) | 6.68 (6.77) | 18.52 (18.77) |
| Non-treated side | 104.70 (25.09) | 104.64 (24.24) | 0.899  0.693 to 0.966 | 7.82  4.17 to 11.47 | 10.89 (10.40) | 7.70 (7.36) | 21.35 (20.40) |
| BIBP – nmastec |  |  |  |  |  |  |  |
| Treated side | 93.20 (23.67) | 103.19 (27.75) | 0.889  0.555 to 0.965 | 9.28  5.89 to 12.67 | 9.80 (9.98) | 8.59 (8.75) | 23.81 (24.25) |
| Non-treated side | 99.94 (26.65) | 104.44 (24.38) | 0.875  0.665 to 0.954 | 7.52  3.67 to 11.37 | 11.97 (11.71) | 8.92 (8.73) | 24.75 (24.22) |
| BIBP – mastec |  |  |  |  |  |  |  |
| Summered | 202.19 (42.13) | 204.52 (44.45) | 0.903  0.708 to 0.967 | 7.35  4.46 to 10.24 | 18.71 (9.20) | 13.26 (6.52) | 36.75 (18.07) |
| BIBP – nmastec |  |  |  |  |  |  |  |
| Summered | 193.15 (48.48) | 207.63 (49.96) | 0.902  0.694 to 0.966 | 7.51  3.89 to 11.13 | 19.13 (9.54) | 15.34 (7.66) | 42.54 (21.23) |
|  |  |  |  |  |  |  |  |
| BP 1-RM - mastec | 21.33 (3.88) | 22.13 (3.31) | 0.969  0.829 to 0.991 | 2.98  0.86 to 5.11 | 0.71 (3.30) | 0.63 (2.89) | 1.74 (8.02) |
| BP 1-RM - nmastec | 24.00 (5.82) | 25.29 (5.10) | 0.945  0.812 to 0.982 | 5.09  1.66 to 8.51 | 1.58 (6.40) | 1.27 (5.17) | 3.53 (14.32) |
|  |  |  |  |  |  |  |  |

HGS, handgrip strength; BIBP, bilateral isometric bench press; *N*, Newton; SD, standard deviation; CV, coefficient of variation; ICC, intra-class coefficient correlation; CI, 95% confidence intervals; SEM, standard error of measurement; MDC, minimally detectable change.

Supplementary material Fig. 3. Bland-Altman plots of maximum muscular strength tests: HGS, handgrip strength; BIBP, bilateral isometric bench press; and, 1-RM, one repetition maximum in BCS with breast cancer surgery mastectomy.


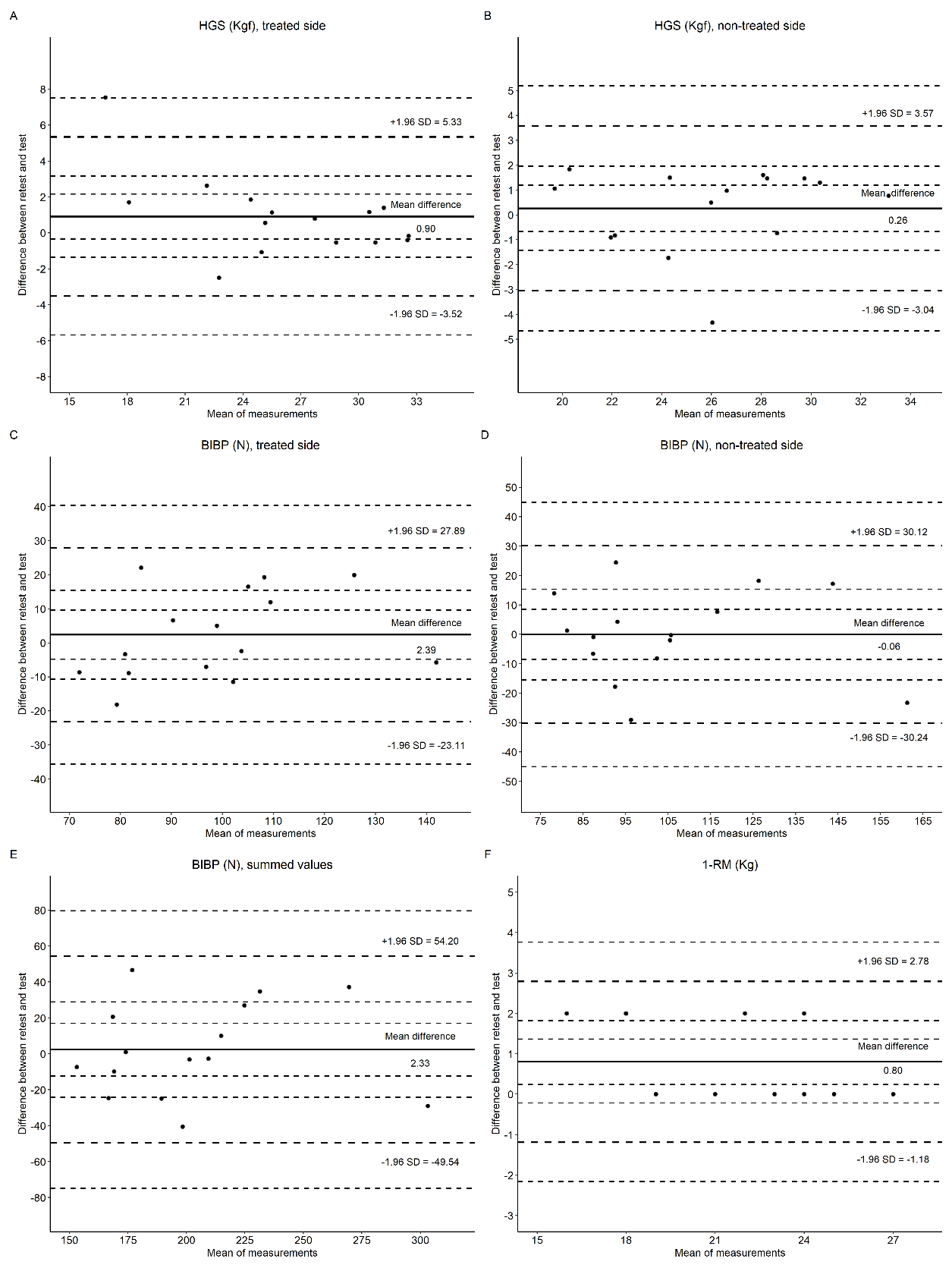


Leg. The dotted line (+1.96 SD and -1.96 SD) represents the limits of agreement between upper and lower boundaries. The continuous line (mean difference) on the center of the plot represents the systematic bias. The continuous line on the Y axis represents the mean difference between retest and test, and the X axis represents the mean of retest and test.

Supplementary material Fig. 4. Bland-Altman plots of maximum muscular strength tests: HGS, handgrip strength; BIBP, bilateral isometric bench press; and, 1-RM, one repetition maximum in BCS without breast cancer surgery mastectomy.


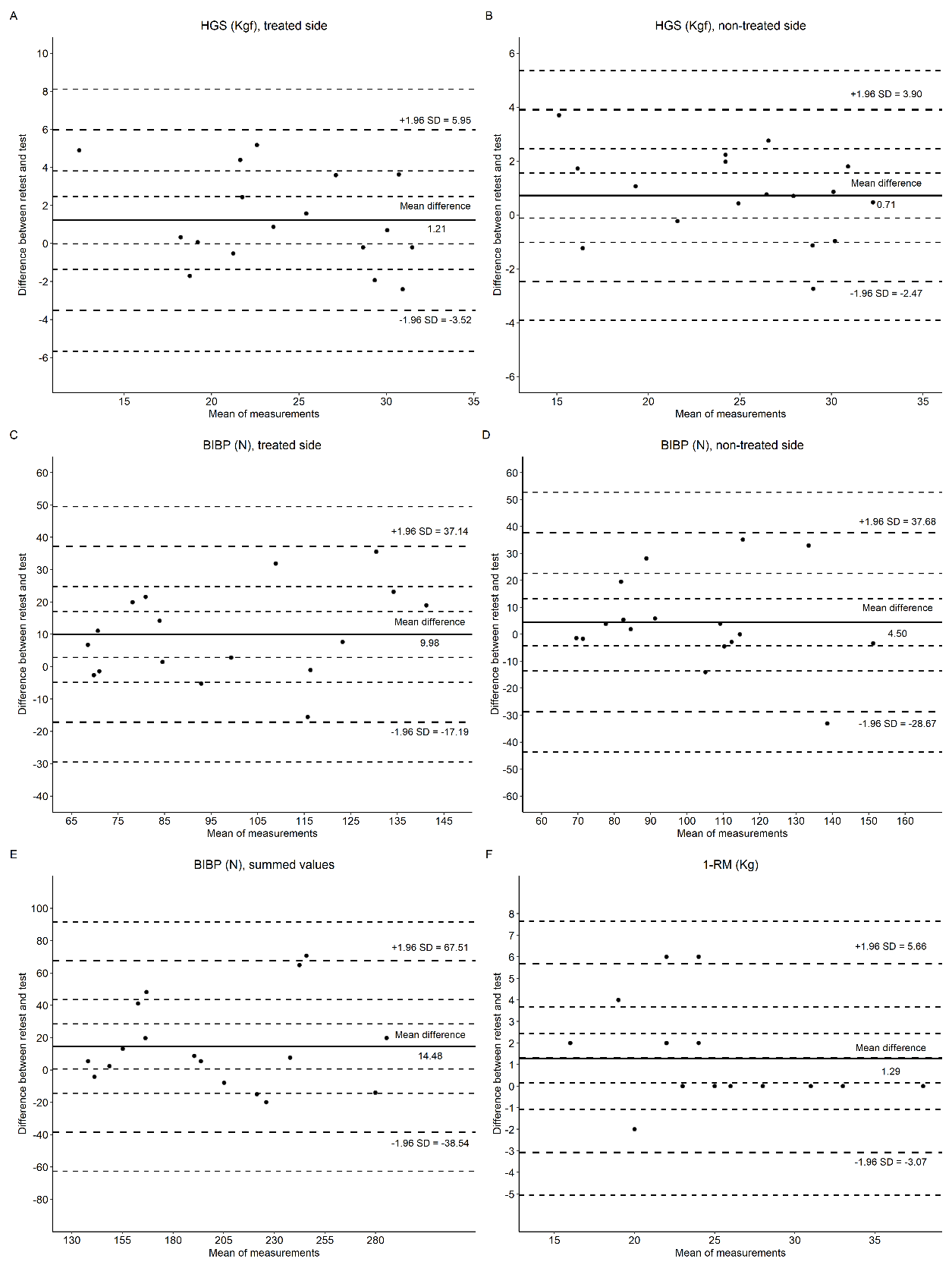


Leg. The dotted line (+1.96 SD and -1.96 SD) represents the limits of the agreement’s upper and lower boundaries. The continuous line (mean difference) on the center of the plot represents the systematic bias. The continuous line on the Y axis represents the mean difference between retest and test, and the X axis represents the mean of retest and test.

**1.2 Supplementary Figures and Tables – Considering upper limb dominance.**

Supplementary material 5. Analysis of intra-day reliability and error of measurement on upper body muscle strength testing in breast cancer survivors considering breast surgery in the dominant side (N = 19) or in the non-dominant side (N = 13).

|  | Attempt 1  Mean (SD) | Attempt 2  Mean (SD) | Attempt 3  Mean (SD) | ICC  95%IC | CV %  95%IC | SEM  (SEM%) | MDC  (MDC%) |
| --- | --- | --- | --- | --- | --- | --- | --- |
| HGS dom - test |  |  |  |  |  |  |  |
| Treated side | 26.52 (4.54) | 26.35 (5.16) | 26.63 (4.56) | 0.978  0.954 to 0.991 | 4.29  2.96 to 5.61 | 0.69 (2.61) | 1.92 (7.23) |
| Non-treated side | 24.84 (5.36) | 25.12 (5.48) | 24.35 (5.28) | 0.985  0.968 to 0.994 | 3.95  2.87 to 5.02 | 0.65 (2.62) | 1.80 (7.26) |
|  |  |  |  |  |  |  |  |
| HGS dom - retest |  |  |  |  |  |  |  |
| Treated side | 26.45 (4.92) | 26.54 (4.93) | 26.82 (4.66) | 0.988  0.975 to 0.995 | 3.08  2.22 to 3.93 | 0.52 (1.97) | 1.45 (5.46) |
| Non-treated side | 25.15 (4.87) | 25.27 (4.66) | 25.43 (4.76) | 0.989  0.977 to 0.995 | 3.14  2.30 to 3.98 | 0.49 (1.95) | 1.37 (5.42) |
|  |  |  |  |  |  |  |  |
| HGS ndom - test |  |  |  |  |  |  |  |
| Treated side | 22.08 (6.48) | 21.99 (7.05) | 22.07 (6.33) | 0.988  0.968 to 0.996 | 5.96  3.37 to 8.55 | 0.72 (3.25) | 1.99 (9.01) |
| Non-treated side | 25.25 (4.86) | 26.09 (4.90) | 25.95 (4.08) | 0.967  0.918 to 0.989 | 4.76  3.00 to 6.51 | 0.81 (3.16) | 2.26 (8.77) |
|  |  |  |  |  |  |  |  |
| HGS ndom -retest |  |  |  |  |  |  |  |
| Treated side | 24.11 (4.92) | 24.64 (4.97) | 24.84 (5.39) | 0.982  0.954 to 0.994 | 3.75  1.59 to 5.92 | 0.67 (2.74) | 1.86 (7.59) |
| Non-treated side | 26.14 (4.99) | 26.11 (4.84) | 26.50 (4.87) | 0.98  0.95 to 0.993 | 3.99  2.47 to 5.51 | 0.68 (2.59) | 1.88 (7.17) |
|  |  |  |  |  |  |  |  |
| BIBP dom - test |  |  |  |  |  |  |  |
| Treated side | 96.42 (20.79) | 96.47 (19.84) | 94.74 (19.89) | 0.982  0.962 to 0.993 | 4.46  3.22 to 5.71 | 2.66 (2.77) | 7.37 (7.69) |
| Non-treated side | 102.84 (26.35) | 102.11 (26.09) | 99.85 (27.04) | 0.981  0.959 to 0.992 | 5.40  3.81 to 7.00 | 3.58 (3.53) | 9.93 (9.78) |
|  |  |  |  |  |  |  |  |
| BIBP dom - retest |  |  |  |  |  |  |  |
| Treated side | 102.13 (25.20) | 104.08 (27.36) | 103.04 (26.32) | 0.982  0.961 to 0.992 | 5.02  3.67 to 6.38 | 3.46 (3.36) | 9.60 (9.32) |
| Non-treated side | 101.47 (25.21) | 104.70 (28.26) | 103.74 (26.03) | 0.977  0.952 to 0.991 | 5.30  3.77 to 6.83 | 3.94 (3.81) | 10.91 (10.56) |
|  |  |  |  |  |  |  |  |
| BIBP ndom - test |  |  |  |  |  |  |  |
| Treated side | 95.22 (23.77) | 94.74 (23.04) | 92.77 (23.66) | 0.983  0.957 to 0.994 | 4.63  2.69 to 6.56 | 3.01 (3.19) | 8.34 (8.85) |
| Non-treated side | 106.52 (30.14) | 101.91 (26.30) | 100.60 (23.22) | 0.974  0.933 to 0.991 | 5.20  2.97 to 7.43 | 4.21 (4.09) | 11.67 (11.33) |
|  |  |  |  |  |  |  |  |
| BIBP ndom -retest |  |  |  |  |  |  |  |
| Treated side | 97.96 (22.54) | 99.84 (25.37) | 100.76 (24.93) | 0.983  0.958 to 0.994 | 4.30  2.91 to 5.69 | 3.12 (3.13) | 8.64 (8.68) |
| Non-treated side | 106.41 (21.57) | 103.59 (20.05) | 109.03 (24.40) | 0.968  0.92 to 0.99 | 4.83  3.11 to 6.54 | 3.84 (3.61) | 10.65 (10.01) |
|  |  |  |  |  |  |  |  |
| BIBP dom |  |  |  |  |  |  |  |
| Summered - test | 199.26 (45.68) | 198.58 (44.42) | 194.59 (45.62) | 0.985  0.968 to 0.994 | 4.12  2.69 to 5.56 | 5.46 (2.76) | 15.13 (7.66) |
| Summered - retest | 203.61 (48.47) | 208.78 (53.78) | 206.78 (51.04) | 0.983  0.964 to 0.993 | 4.40  3.09 to 5.70 | 6.56 (3.18) | 18.17 (8.81) |
|  |  |  |  |  |  |  |  |
| BIBP ndom |  |  |  |  |  |  |  |
| Summered - test | 201.74 (52.14) | 196.64 (47.82) | 193.38 (45.35) | 0.982  0.955 to 0.994 | 4.41  2.53 to 6.29 | 6.40 (3.25) | 17.75 (9.00) |
| Summered - retest | 204.37 (40.87) | 203.43 (43.19) | 209.78 (46.95) | 0.981  0.953 to 0.994 | 3.97  2.67 to 5.26 | 5.92 (2.88) | 16.42 (7.98) |

HGS, handgrip strength; BIBP, bilateral isometric bench press; dom, dominant; ndom, non-dominant *N*, Newton; SD, standard deviation; CV, coefficient of variation; ICC, intra-class coefficient correlation; CI, 95% confidence intervals; SEM, standard error of measurement; MDC, minimally detectable change.

Supplementary material 6. Analysis of test-retest reliability and error of measurement on upper body muscle strength testing in breast cancer survivors considering breast surgery in the dominant side (N = 19) or in the non-dominant side (N = 13).

|  | Test  Mean (SD) | Retest  Mean (SD) | ICC  95%CI | CV (%)  95%CI | TEM (TEM%) | SEM (SEM%) | MDC (MDC%) |
| --- | --- | --- | --- | --- | --- | --- | --- |
| HGS – dom |  |  |  |  |  |  |  |
| Treated side | 26.5 (4.66) | 26.60 (4.78) | 0.976  (0.938 to 0.991) | 3.01  (1.68 to 4.34) | 1.04 (4.19) | 0.82 (3.30) | 2.28 (9.16) |
| Non-treated side | 24.77 (5.30) | 25.28 (4.71) | 0.97  (0.923 to 0.988 | 4.31  (2.35 to 6.27) | 1.18 (4.77) | 0.77 (3.11) | 2.14 (8.63) |
| HGS – ndom |  |  |  |  |  |  |  |
| Treated side | 22.05 (6.54) | 24.53 (5.01) | 0.907  (0.38 to 0.977) | 10.43  4.60 to 16.27 | 1.86 (7.22) | 1.69 (6.57) | 4.69 (18.21) |
| Non-treated side | 25.76 (4.48) | 26.25 (4.80) | 0.967  (0.842 to 0.994) | 4.23  (2.93 to 5.53) | 1.18 (4.47) | 0.94 (3.58) | 2.61 (9.93) |
| BIBP – dom |  |  |  |  |  |  |  |
| Treated side | 95.88 (19.83) | 103.08 (25.83) | 0.882  (0.658 to 0.957) | 8.90  (6.23 to 11.58) | 9.75 (9.75) | 7.20 (7.21) | 19.97 (19.98) |
| Non-treated side | 101.60 (26.00) | 103.30 (25.96) | 0.918  (0.787 to 0.968) | 6.61  (3.65 to 9.57) | 10.30 (10.04) | 6.52 (6.35) | 18.06 (17.61) |
| BIBP – ndom |  |  |  |  |  |  |  |
| Treated side | 94.24 (23.09) | 99.52 (23.91) | 0.893  (0.662 to 0.967) | 8.32  (4.13 to 12.51) | 10.11 (10.51) | 8.44 (8.77) | 23.38 (24.31) |
| Non-treated side | 103.01 (26.11) | 106.34 (21.48) | 0.825  (0.428 to 0.946) | 9.42  (4.26 to 14.59) | 13.27 (12.71) | 11.52 (11.03) | 31.94 (30.58) |
| BIBP – dom |  |  |  |  |  |  |  |
| Summered | 197.48 (44.58) | 206.39 (50.29) | 0.926  (0.808 to 0.972) | 6.70  (4.18 to 9.23) | 17.00 (8.39) | 11.39 (5.62) | 31.56 (15.58) |
| BIBP – ndom |  |  |  |  |  |  |  |
| Summered | 197.26 (47.73) | 205.86 (42.98) | 0.859  (0.554 to 0.956) | 8.51  (4.04 to 12.99) | 22.63 (11.28) | 19.46 (9.70) | 53.94 (26.88) |
|  |  |  |  |  |  |  |  |
| BP 1-RM - dom | 22.95 (6.16) | 24.00 (5.42) | 0.971  (0.896 to 0.99) | 3.84  (1.02 to 6.67) | 1.19 (5.15) | 0.88 (3.83) | 2.45 (10.61) |
| BP 1-RM - ndom | 22.46 (3.23) | 23.54 (3.18) | 0.879  (0.576 to 0.964) | 4.48  (1.30 to 7.66) | 1.37 (5.81) | 1.52 (6.46) | 4.22 (17.92) |
|  |  |  |  |  |  |  |  |

HGS, handgrip strength; BIBP, bilateral isometric bench press; *N*, Newton; dom, dominant; ndom, non-dominant; SD, standard deviation; CV, coefficient of variation; ICC, intra-class coefficient correlation; CI, 95% confidence intervals; TEM, typical error of measurement; SEM, standard error of measurement; MDC, minimally detectable change.


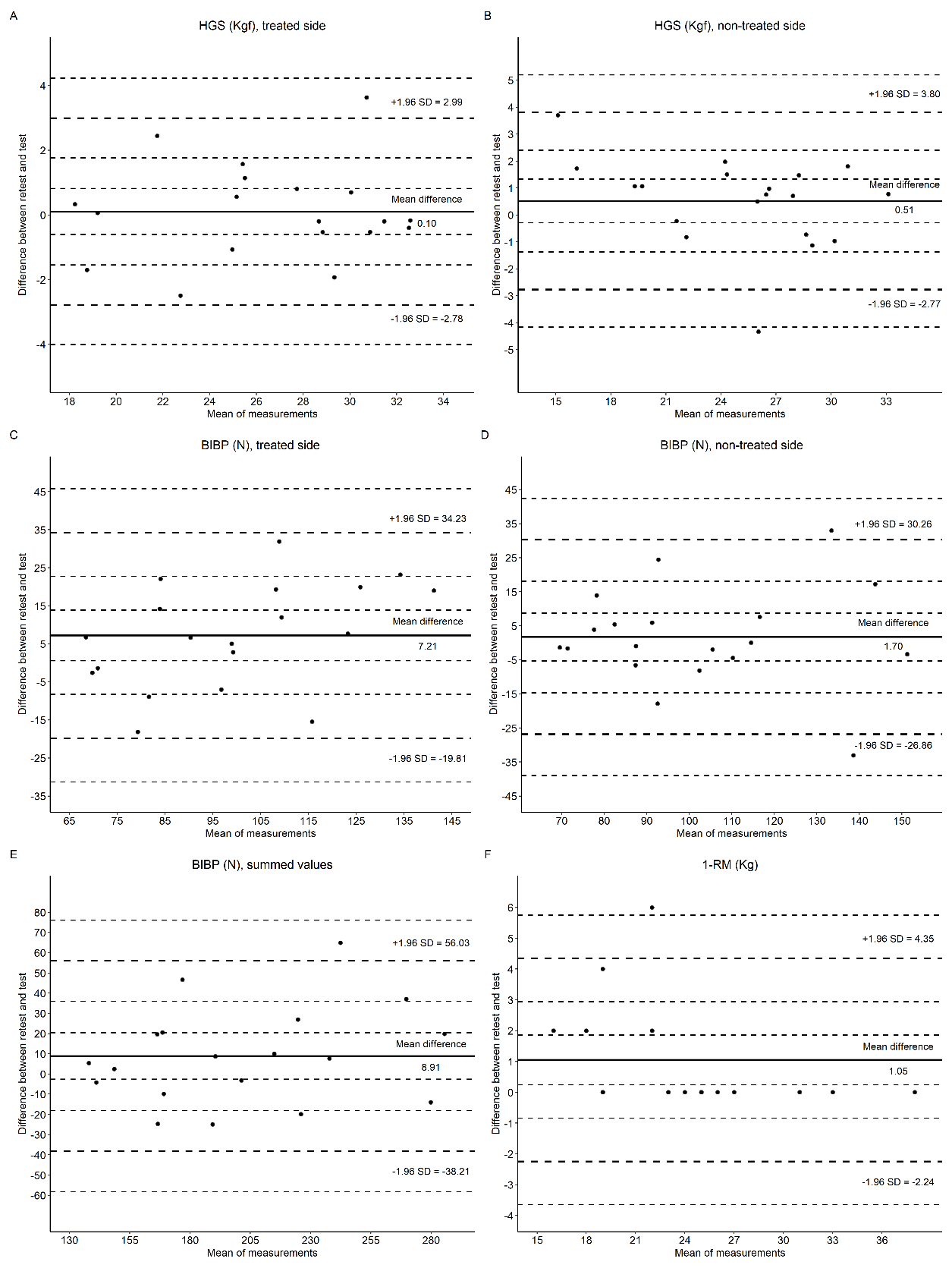
Supplementary material Fig. 5. Bland-Altman plots of maximum muscular strength tests: HGS, handgrip strength; BIBP, bilateral isometric bench press; and, 1-RM, one repetition maximum in BCS when breast cancer surgery occurred in the dominant side.

Leg. The dotted line (+1.96 SD and -1.96 SD) represents the limits of agreement between upper and lower boundaries. The continuous line (mean difference) on the center of the plot represents the systematic bias. The continuous line on the Y axis represents the mean difference between retest and test, and on the X axis represents the mean of retest and test.


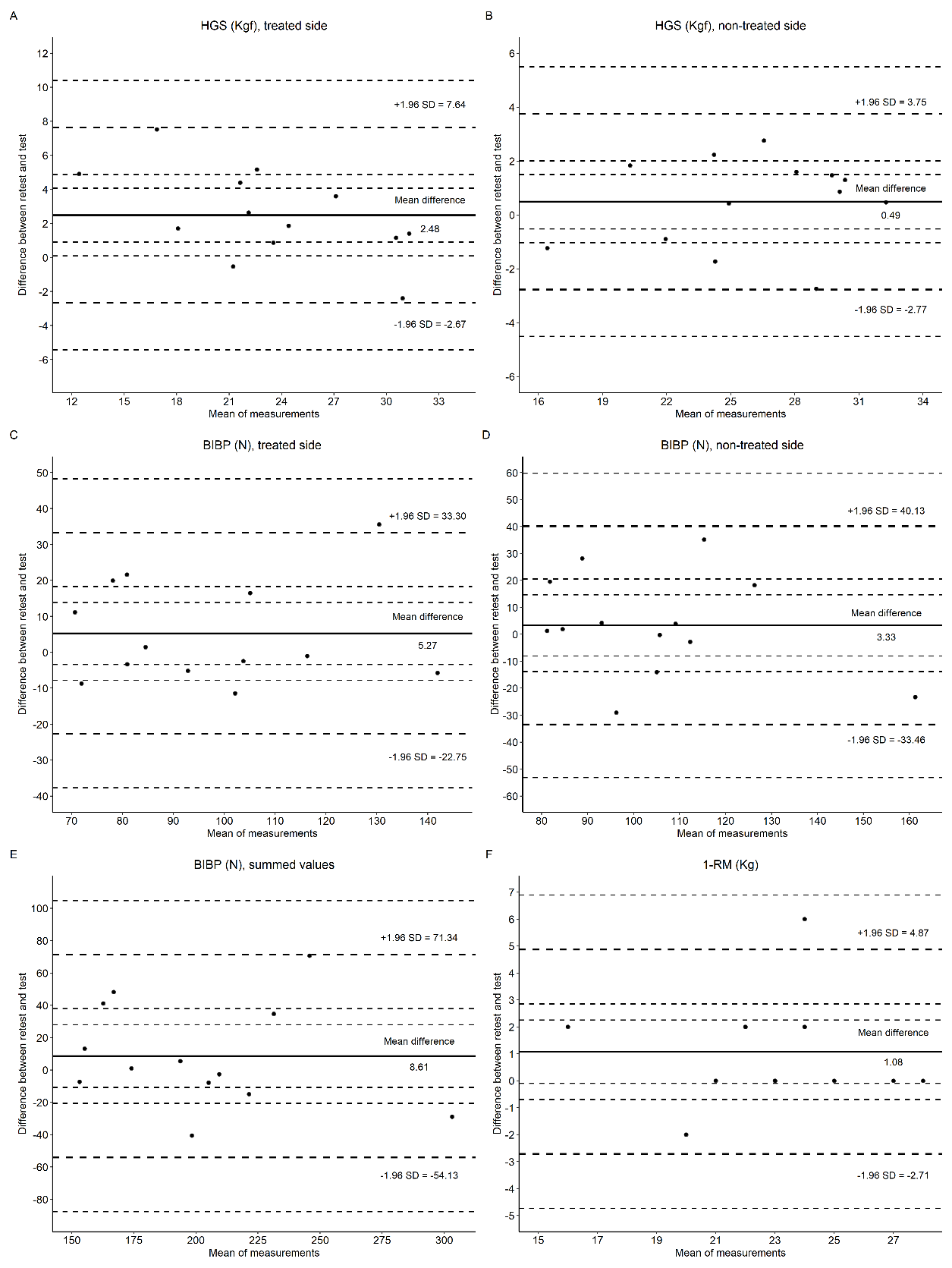
Supplementary material Fig. 6. Bland-Altman plots of maximum muscular strength tests: HGS, handgrip strength; BIBP, bilateral isometric bench press; and, 1-RM, one repetition maximum in BCS when breast cancer surgery occurred in the non-dominant side.

Leg. The dotted line (+1.96 SD and -1.96 SD) represents the limits of agreement between upper and lower boundaries. The continuous line (mean difference) on the center of the plot represents the systematic bias. The continuous line on the Y axis represents the mean difference between retest and test, and on the X axis represents the mean of retest and test.
